# NfL and pNfH are increased in Friedreich’s ataxia

**DOI:** 10.1007/s00415-020-09722-6

**Published:** 2020-01-30

**Authors:** Stefanie Nicole Hayer, Inga Liepelt, Christian Barro, Carlo Wilke, Jens Kuhle, Peter Martus, Ludger Schöls, the EFACTS study group

**Affiliations:** 1 Department of Neurodegenerative Diseases, Hertie-Institute for Clinical Brain Research and Center of Neurology, University of Tuebingen, Hoppe-Seyler-Str. 3, 72076 Tuebingen, Germany; 2 German Research Center for Neurodegenerative Diseases (DZNE), Tuebingen, Germany; 3 Neurologic Clinic and Policlinic, Departments of Medicine, Biomedicine and Clinical Research, University Hospital Basel, University of Basel, Basel, Switzerland; 4 Institute for Clinical Epidemiology and Applied Biometry, University of Tuebingen, Tuebingen, Germany

**Keywords:** Neurofilament light chain, NfL, Friedreich’s ataxia, Biomarker, Neurofilament heavy chain, pNfH

## Abstract

**Objective:**

To assess neurofilaments as neurodegenerative biomarkers in serum of patients with Friedreich’s ataxia.

**Methods:**

Single molecule array measurements of neurofilament light (NfL) and heavy chain (pNfH) in 99 patients with genetically confirmed Friedreich’s ataxia. Correlation of NfL/pNfH serum levels with disease severity, disease duration, age, age at onset, and GAA repeat length.

**Results:**

Median serum levels of NfL were 21.2 pg/ml (range 3.6–49.3) in controls and 26.1 pg/ml (0–78.1) in Friedreich’s ataxia (*p* = 0.002). pNfH levels were 23.5 pg/ml (13.3–43.3) in controls and 92 pg/ml (3.1–303) in Friedreich’s ataxia (*p* = 0.0004). NfL levels were significantly increased in younger patients (age 16–31 years, *p* < 0.001) and patients aged 32–47 years (*p* = 0.008), but not in patients of age 48 years and older (*p* = 0.41). In a longitudinal assessment, there was no difference in NfL levels in 14 patients with repeated sampling 2 years after baseline measurement. Levels of NfL correlated inversely with GAA1 repeat length (*r* = − 0.24, *p* = 0.02) but not with disease severity (*r* = − 0.13, *p* = 0.22), disease duration (*r *= − 0.06, *p* = 0.53), or age at onset (*r* = 0.05, *p* = 0.62).

**Conclusion:**

Serum levels of NfL and pNfH are elevated in Friedreich’s ataxia, but differences to healthy controls decrease with increasing age. Long-term longitudinal data are required to explore whether this reflects a selection bias from early death of more severely affected individuals or a slowing down of the neurodegenerative process with age. In a pilot study over 2 years of follow-up—a period relevant for biomarkers indicating treatment effects—we found NfL levels to be stable.

**Electronic supplementary material:**

The online version of this article (10.1007/s00415-020-09722-6) contains supplementary material, which is available to authorized users.

## Introduction

Friedreich’s ataxia is the most frequent type of autosomal recessive ataxia in the western world with a prevalence of about 1:36,000 [1]. In most cases, it is caused by homozygous GAA repeat expansions in the first intron of the *FXN* gene that result in reduced levels of frataxin and in iron/sulfur clusters leading to disturbance of e. g. the respiratory chain [2]. The progressive nature of Friedreich’s ataxia leads to continuous destruction of neurons with a focus on long fibre tracts in the spinal cord, causing progressive degeneration of dorsal root ganglia, posterior columns, sensory nerves, and corticospinal tracts [3]. During this process, axonal cytoskeletal proteins are likely to be liberated into cerebral spinal fluid (CSF) and even into blood, in consequence of neuro-axonal injury. An integral component of the axonal cytoskeleton is neurofilament light chain (NfL) and phosphorylated neurofilament heavy chain (pNfH). These markers were recently shown to be increased in the cerebrospinal fluid of several progressive neurodegenerative diseases including amyotrophic lateral sclerosis (ALS), frontotemporal dementia (FTD), Creutzfeldt–Jakob disease (CJD), and adult-onset leukoencephalopathy with axonal spheroids (ALSP) [4–9]. Most lately, ultrasensitive assays allow assessments of NfL and pNfH also in serum [10, 11].

Biomarkers reflecting degenerative processes are important for interventional trials that aim to slow disease progression. In Friedreich’s ataxia, frataxin protein level is used as a biomarker reflecting an early event in pathophysiology, as it was shown to be reduced in patients in consequence of the intronic repeat expansion leading to impaired transcription of the *FXN* gene [2]. As lack of frataxin is thought to be the pathomechanism driving all further steps in the pathogenesis of Friedreich’s ataxia, several therapeutic approaches are aiming to increase transcription of the *FXN* gene. In these studies, frataxin levels are monitored to document treatment success [12, 13]. However, serum markers reflecting the degenerative aspects of the disease are missing.

Here, we assessed serum levels of NfL and pNfH in patients with Friedreich’s ataxia and healthy controls by ultrasensitive single molecule array (Simoa) [14, 15] using a cross-sectional and a longitudinal approach.

## Methods

### Subjects

A total of 99 patients (median age 38 years, range 16–68) with genetically confirmed Friedreich’s ataxia were recruited through the European Friedreich’s Ataxia Consortium for Translational Studies (EFACTS). All patients carried GAA repeat expansions on both alleles. Repeat lengths of the shorter allele varied between 67 and 1167 GAA repeats and on the longer allele between 200 and 1500 repeats. We assessed clinical disease severity with the Scale for the Assessment and Rating of Ataxia (SARA), a validated score that allows to quantify an individuals’ degree of ataxia and ranges from 0 (no ataxia) to 40 points (most severe ataxia) [16]. Age at onset was defined by first reported symptoms, and disease duration as the period between onset and time of sampling. In addition, 30 individuals (median age 48 years, range 18–68) were enrolled at the Department of Neurodegenerative Disorders, Hertie Institute for Clinical Brain Research, University Hospital Tübingen, as healthy controls. All controls were assessed by neurologists with special expertise in neurodegenerative diseases, ascertaining that none of them had a history or clinical signs of neurodegenerative disease or of any other major neurological disorder. The study has been approved by the institutional review board, and all subjects gave written informed consent prior to participation.

### Biomaterial

The biomaterial from Friedreich’s ataxia patients used in this study was provided by the centralized biomaterial bank of the medical faculty of the RWTH Aachen University (RWTH cBMB) and used in accordance with the biomaterial bank’s regulations and vote 206/09 of the ethics committee of the medical faculty of the RWTH Aachen University. Serum samples from healthy controls were provided by the biobank of the Hertie Institute for Clinical Brain Research (HIH), University of Tübingen, and used in accordance with the biomaterial bank’s regulations and vote 199/2011BO1 of the ethics committee of the medical faculty of the University of Tübingen. Samples were frozen at − 80 °C within 90 min after collection, and analysed without any previous thaw–freeze cycle.

### Measurements

NfL concentrations were analysed by single molecule array (Simoa) assay as previously described [11]. Inter-assay coefficients of variation (CV) for three native serum samples were 14.6%, 7.5%, and 2.1% for control samples with mean concentrations of 7.7 pg/ml, 22.6 pg/ml, and 77.4 pg/ml, respectively. The mean intra-assay CV of duplicate determinations for concentration was 3.7%. pNfH was quantified by a commercially available Kit (Quanterix) on the Simoa platform on a single run. The mean intra-assay CV of duplicate determinations for concentration was 9.5%.

### Statistical analysis

Statistics were performed using nonparametric tests in bivariate analysis. Multiple regression models were used to assess dependency of NfL on age. Data in the results section report median and range, data in graphs represent median and 95% confidence interval (CI). Demographic data were compared using two-tailed Mann–Whitney test. For pNfH, the Mann–Whitney *U *test was used to compare values between patients and controls. A two-tailed Wilcoxon test was applied to compare paired data from longitudinal measurements in identical subjects. Correlation was assessed by computing Spearman r. One outlier was removed in the pNfH control group (pNfH = 346.1 pg/ml), by the robust regression and outlier removal (ROUT) method (*Q* = 0.1%) in GraphPad Prism 7. To assess a possible use of NfL measurements for diagnosis, an age-corrected ROC curve was constructed. This was done by first calculating a quadratic regression model for NfL dependent on age in the control group only. Second, the equation of this model was used to calculate predicted values of NfL for both, the control and the patient group. Third, the differences between observed and predicted values of NfL were calculated and used as continuous classificator in a ROC analysis. This led to the “age-corrected ROC curve”. Regression and ROC analyses were performed using SPSS 25 for Windows. All other analyses were performed with GraphPad Prism 7 for Mac.

## Data availability

Anonymized data will be shared on request of qualified investigators.

## Results

### NfL and pNfH mark neuronal damage in Friedreich’s ataxia

**Fig. 1:**
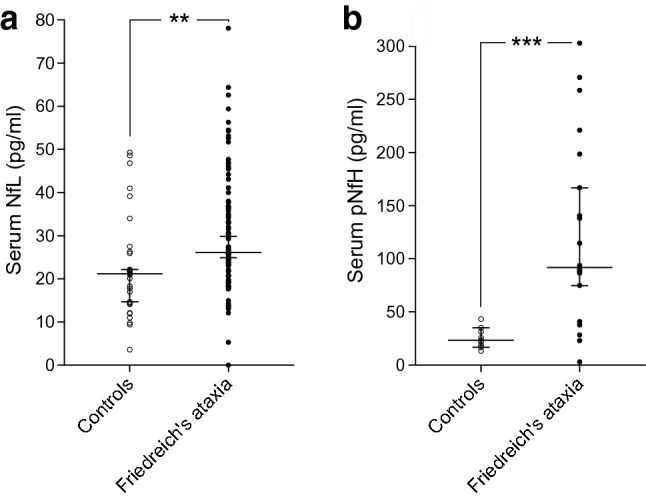
NfL and pNfH are increased in Friedreich’s ataxia**. a** Serum NfL of 30 healthy controls and 99 genetically confirmed Friedreich’s ataxia patients. **b** Serum pNfH in 9 healthy controls and 20 patients with Friedreich’s ataxia. **p = 0.0019, ***p = 0.0004. Data represent median and 95% confidence interval. *NfL* neurofilament light chain, *pNfH* phosphorylated neurofilament heavy chain

To investigate whether serum NfL and pNfH might serve as biomarkers in Friedreich’s ataxia, we compared NfL serum levels between 30 healthy controls and 99 patients with Friedreich’s ataxia as well as pNfH serum levels in a subgroup of 9 controls and 20 patients. The median NfL concentration in controls was 21.15 pg/ml (range 3.6–49.3), while the concentration in Friedreich’s ataxia was significantly higher with 26.1 pg/ml (range 0–78.1; *p* = 0.002) (Fig. 1a). Similarly, pNfH levels were significantly elevated in patients compared to controls (controls 23.5 pg/ml, range 13.3–43.2; Friedreich’s ataxia 92 pg/ml, range 3.1–303; *p* = 0.0004) (Fig. 1b).

**Fig. 2:**
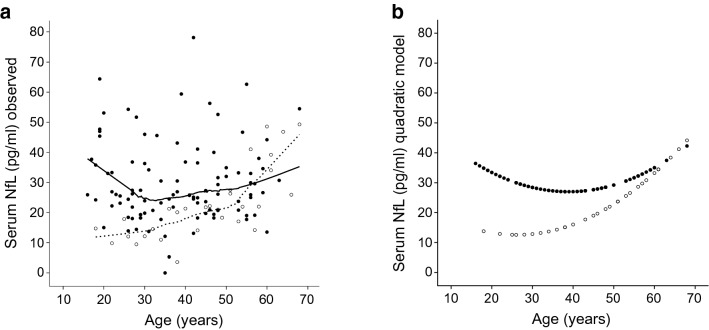
NfL levels in healthy controls (white dots) and Friedreich’s ataxia (black dots) relative to age. Serum NfL allows an excellent classification in *healthy* or *affected* for younger individuals (16–31 years), a moderately good classification for middle aged individuals (32–47 years), and no classification better than chance for older individuals (48–68 years). **a** Observed NfL values with LOWESS fit (dashed line for controls and continuous line for patients). **b** Calculated NfL values with quadratic fit. *NfL* neurofilament light chain

Controls (45.27 ± 14.11 years) were older than patients with Friedreich’s ataxia (38.37 ± 13.05 years; *p* = 0.02), but covered a comparable age range (controls 18–68 years, patients 16–68 years). Thus, age dependency of NfL was assessed in detail for both groups. In healthy individuals, there was a clear quadratic dependency of NfL on age (Fig. 2a, dashed line and Supplementary Figure S1a): in controls aged < 30 years, there was no increase of NfL with age. Between 30 and 50 years of age, there was a moderate increase and in the age range of 50–65, there was a steep increase (*r*-square linear model = 0.55, quadratic model 0.64, both *p* < 0.00001). In diseased subjects, no significant age dependency of NfL levels could be detected with a non-significant trend to a quadratic model (*r*-square linear model = 0.00, *p* = 0.90, quadratic model 0.05, *p* = 0.095). The area under the age-corrected ROC curve (cf. “Methods” section) was 0.78 (95% CI 0.69–0.86). Separate analysis within three equally sized strata of age (16–31 years, 32–47 years, 48–68 years) revealed areas under the ROC curve of 0.99 (CI 0.95–1.00, *p* < 0.001), 0.81 (0.70–0.92, *p* = 0.002) and 0.49 (0.28–0.70, *p* = 0.94). Thus, based on NfL measurements, there was an excellent classification in *healthy* or *affected* for younger individuals, a moderately good classification for middle aged individuals and no classification better than chance for older individuals.

### Serum NfL is increased independently of disease severity, age at onset, and disease duration in Friedreich’s ataxia

**Fig. 3:**
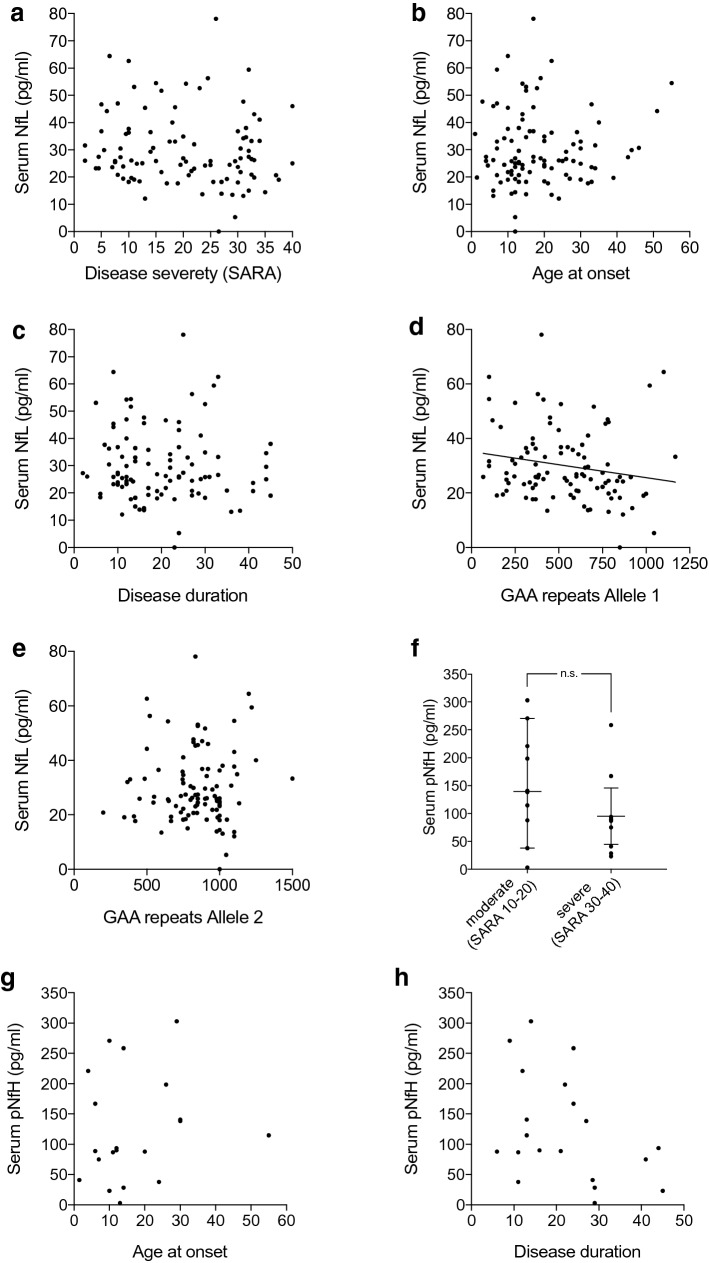
NfL and pNfH do not correlate with disease severity, age at onset or disease duration in Friedreich’s ataxia. **a** Serum NfL levels versus disease severity in Friedreich’s ataxia, quantified by SARA. **b** Correlation of serum NfL levels with age at symptom onset. **c** Serum NfL versus disease duration, defined by the interval between first reported symptoms and blood sampling. **d** Correlation of serum NfL levels with the repeat length of the shorter allele and **e** with the longer allele. **f** Quantification of serum pNfH levels versus disease severity in patients with Friedreich’s ataxia that were a priori categorized as moderately (SARA 10–20) or severely (SARA 30–40) affected. **g** Correlation of pNfH with age at onset, and **h** with disease duration. Data in **f** represent median and 95% confidence interval. A linear regression line is only depicted in graphs presenting a statistically significant correlation. *NfL* neurofilament light chain, *pNfH* phosphorylated neurofilament heavy chain, *SARA* scale for the assessment and rating of ataxia, *ns* not significant

We assessed serum NfL levels in correlation with disease severity as defined by the Scale for the Assessment and Rating of Ataxia (SARA) score. We found that serum NfL did not correlate with the SARA score (*r* = − 0.13, 95% CI − 0.32 to 0.08, *n* = 99; *p* = 0.22) (Fig. 3a). Similarly, there was no correlation between NfL concentration and age at onset (*r* = 0.05, 95% CI − 0.15 to 0.25, *n* = 99; *p* = 0.62) (Fig. 3b) or disease duration (*r* = − 0.06, 95% CI − 0.26 to 0.14, *n* = 99; *p* = 0.53) (Fig. 3c). In patients, there was a small, but significant inverse correlation between levels of NfL and the length of the smaller GAA repeat (allele 1) (*r* = − 0.24; 95% CI − 0.42 to − 0.03; *n *= 99, *p* = 0.02) (Fig. 3d), but not with the GAA repeat length in the larger allele (allele 2) (*r* = − 0.01; 95% CI − 0.21 to 0.20, *n* = 99; *p* = 0.95) (Fig. 3e).

Serum pNfH levels were measured in a subgroup of 20 patients that were selected a priori to represent moderately (SARA score 10–20) or severely affected (SARA score 30–40) individuals, to increase the visibility of potential differences from disease severity despite the small group size. Analogous to NfL, there was no correlation between pNfH level and SARA score (*r* = − 0.20, 95% CI − 0.60 to 0.28; *n* = 20, *p* = 0.41) (Supplementary Figure S2). Interestingly, there was even a tendency towards lower pNfH levels in more severely affected patients, albeit this tendency did not reach significance (SARA 10–20: 139.5 pg/ml, range 3.1–303, *n* = 10; SARA 30–40: 87.75 pg/ml, range 23.1–258.6, *n* = 10; *p* = 0.17) (Fig. 3f). pNfH also did not correlate with age, neither in controls nor in Friedreich’s ataxia patients (controls *r* = 0.10, *p* = 0.80, *n* = 9; patients *r* = − 0.11, 95% CI − 0.54 to 0.36, *n* = 20; *p* = 0.64) (Supplementary Figure S1c, d) or age at onset (*r* = 0.18, 95% CI − 0.30 to 0.59, *n* = 20; *p* = 0.44) (Fig. 3g). There were small, but non-significant correlations between pNfH concentration and disease duration (*r* = − 0.39, 95% CI − 0.72 to 0.08, *n* = 20; *p* = 0.09) (Fig. 3h) and also with GAA repeat length of allele 1 (*r* = − 0.32, 95% CI − 0.68 to 0.16, *n* = 20; *p* = 0.17), but not with repeat length of allele 2 (*r* = 0.06, 95% CI − 0.41 to 0.50, *n* = 20; *p* = 0.81) (Supplementary Figure S3).

### Serum NfL remains stable over 2 years in Friedreich’s ataxia

**Fig. 4:**
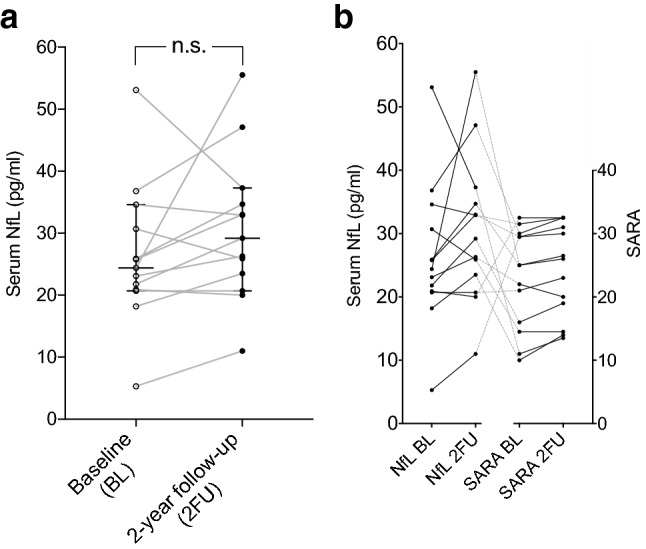
Longitudinal NfL levels in Friedreich’s ataxia. **a** NfL levels and level dynamics of individual patients over a period of 2 years. **b** Comparison of NfL level dynamics and SARA dynamics in individual patients over a period of 2 years. Data in **a** represent median and 95% confidence interval. *BL* baseline, *2FU* 2-year follow-up, *NfL* neurofilament light chain, *SARA* scale for the assessment and rating of ataxia, *ns* not significant

To assess progression dynamics of NfL in Friedreich’s ataxia, we used a longitudinal approach in a group of 14 patients by measuring serum NfL at baseline (BL) and 2 years later (2FU). On individual level, we observed an increase of serum NfL in 9 of 14 patients (64.3%) while concentrations decreased in 4 patients (28.6%) and stayed the same in one patient (Fig. 4a). Overall, there was no significant change during the 2-year period (BL 27.5 pg/ml, range 5.3–53.1; 2FU 34.1 pg/ml, range 11–80.8; *n* = 14, *p* = 0.06) (Fig. 4a). While there was a significant increase in the SARA score over time (BL 21.7 points, range 6–32.5; 2FU 23.5 points, range 13.5–32.5, *n* = 14; *p* = 0.007) (Supplementary Figure S4), the individual dynamics of NfL (increase/decrease) did not match the dynamics of the SARA score (Fig. 4b), congruent to the lack of correlation between NfL levels and disease severity measured by SARA (see Fig. 3c).

## Discussion

This systematic analysis of neurofilament levels in Friedreich’s ataxia demonstrates that NfL and pNfH are significantly elevated in patients with Friedreich’s ataxia. Moreover, we explored possible age effects and could show that NfL levels are an excellent classifier for younger, but not for older patients. The difference we found in the amount of detected NfL between Friedreichs’s ataxia patients and controls compares well with other slowly progressing neurodegenerative diseases such as hereditary spastic paraplegia, Alzheimer’s disease, and spinocerebellar ataxia [17–19]. Levels are lower than in rapidly progressing neurodegenerative diseases such as ALS, FTD, CJD or ALSP [4–7, 9], most likely reflecting the slower decay of axons, with less neurofilament liberated into CSF and peripheral blood per unit of time.

On first glance, it may be surprising that neurofilament levels did not correlate with disease severity, age at onset or disease duration in Friedreich’s ataxia. However, the lack of correlation with disease duration is likely to reflect a rather linear and not exponential course of (axonal) degeneration in Friedreich’s ataxia.

Interestingly, NfL levels in Friedreich’s ataxia lack an age effect as it is observed in healthy controls (Supplementary Figures S1a, b) and well known from the literature [20]. This may indicate that axonal degeneration does not increase with age in Friedreich’s ataxia, but takes place already earlier in life and runs with a continuous rate. The lack of age dependence of NfL levels in Friedreich’s ataxia may even reflect a decrease of the disease specific neurodegeneration with age if the normal age-dependent increase of NfL is taken into account. Alternatively, it reflects a selection bias from mortality that prevented patients with more aggressive courses of Friedreich’s ataxia to reach older age. In respect to therapeutic interventions, it therefore needs to be discussed if NfL is able to indicate the degenerative process of Friedreich’s ataxia in elder patients. Data from a more rapid neurodegenerative disorder, ALS, indicated that NfL levels decrease in later stages, probably because the majority of axons has been lost earlier [20].

A longitudinal assessment of NfL levels in Friedreich’s ataxia is still missing. Our study provides 2-year follow-up data in a small subsample, but did not show a significant change over a timespan that is relevant for interventional trials. In accordance, we found NfL levels to be similar in all disease stages concerning disease severity as well as disease duration.

We found an inverse correlation of NfL levels with GAA repeat length in the smaller allele indicating lower levels of NfL in patients with longer repeat expansions. This finding is difficult to interpret as larger repeat expansions go along with earlier onset in Friedreichs’ ataxia [21] and are supposed to lead to a more severe course of the disease that is expected to result—if anything—in higher levels of NfL. Since we did not find a correlation of NfL with age of onset or disease severity, we suggest to regard the correlation of GAA repeat length of allele 1 and NfL with caution and await its reproduction in an independent cohort.

pNfH is an integral part especially of large, myelinated axons, which are severely affected in Friedreich’s ataxia [22]. To determine whether pNfH is advantageous over NfL, we performed additional pNfH measurements. Indeed, the elevation of pNfH in Friedreich’s ataxia was even more pronounced than the elevation of NfL, and showed only minimal overlap with the control group in our pilot study.

As NfH becomes phosphorylated post-translationally while being transported from the neuronal cell soma in the axon, the higher level of pNfH compared to NfL may reflect an imbalance between regenerative and degenerative processes in the nervous system and the effort to maintain axonal integrity [23–25].

Larger cohorts should be investigated in the future including longitudinal assessments of pNfH serum levels to confirm the more prominent elevation of pNfH and evaluate its potential as disease monitoring marker in Friedreich’s ataxia.

One limitation of this study is the limited number of controls and the age difference between patients and the control group. As NfL is well established to increase with age [26] (Figure S1a), age differences are likely to lead to an underestimate of group differences in our study. However, similar data have been obtained from a recent pilot study with a small group of patients and age-matched controls [27]. As our study was limited to patients older than 16 years of age, it will be of interest to investigate a younger group of patients with longer repeats and shorter disease duration.

In conclusion, we propose NfL and pNfH as new biomarkers that reflect the neurodegenerative process in long fibre tracts in Friedreich’s ataxia. Long-term longitudinal data are required to explore whether the approximation of NfL levels between the Friedreich group and healthy controls with increasing age reflects a selection bias from early death of more severely affected patients or a slowing down of the neurodegenerative process in Friedreich’s ataxia over the course of disease. Our study proposes neurofilaments as potential biomarkers for the assessment of neurodegeneration in interventional trials that aim to slow down disease activity in Friedreich’s ataxia.

### Electronic supplementary material

Below is the link to the electronic supplementary material.

Supplementary file1 (DOCX 132 kb)

## Appendix 1: Authors

**Table:**

Stefanie N. Hayer	University of Tuebingen, Germany	Author
Inga Liepelt	University of Tuebingen, Germany	Author
Christian Barro	University of Basel, Switzerland	Author
Carlo Wilke	University of Tuebingen, Germany	Author
Jens Kuhle	University of Basel, Switzerland	Author
Peter Martus	University of Tuebingen, Germany	Author
Ludger Schöls	University of Tuebingen, Germany	Author

## Appendix 2: Co-investigators of the European Friedreich’s Ataxia Consortium for Translational Studies (EFACTS)

**Table:**

Schulz, Jörg Bernhard	Department of Neurology, RWTH Aachen University; JARA-BRAIN Institute Molecular Neuroscience and Neuroimaging, Forschungszentrum Jülich GmbH and RWTH Aachen University, Germany	Principle investigator, Program coordinator
Reetz, Kathrin	Department of Neurology, RWTH Aachen University; JARA-BRAIN Institute Molecular Neuroscience and Neuroimaging, Forschungszentrum Jülich GmbH and RWTH Aachen University, Germany	Site investigator
Fedosov, Kathrin	Department of Neurology, RWTH Aachen University, Germany	Database oversight
Didszun, Claire	Department of Neurology, RWTH Aachen University, Germany	Database oversight
Klockgether, Thomas	Department of Neurology, University Hospital of Bonn; German Center for Neurodegenerative Diseases (DZNE), Bonn, Germany	Principle investigator
Giordano, Ilaria	Department of Neurology, University Hospital of Bonn; German Center for Neurodegenerative Diseases (DZNE), Bonn, Germany	Site investigator
Pandolfo, Massimo	Laboratory of Experimental Neurology, Université Libre de Bruxelles, Brussels, Belgium	Principle investigator
Depondt, Chantal	Laboratory of Experimental Neurology, Université Libre de Bruxelles, Brussels, Belgium	Site investigator
Rai, Myriam	Laboratory of Experimental Neurology, Université Libre de Bruxelles, Brussels, Belgium	Genetics
Boesch, Sylvia	Department of Neurology, Medical University Innsbruck, Austria	Principle investigator
Nachbauer, Wolfgang	Department of Neurology, Medical University Innsbruck, Austria	Site investigator
Eigentler, Andreas	Department of Neurology, Medical University Innsbruck, Austria	Site investigator
Indelicato, Elisabetta	Department of Neurology, Medical University Innsbruck, Austria	Site investigator
Giunti, Paola	Department of Molecular Neuroscience, Institute of Neurology, University College London, London, UK	Principal investigator
Parkinson, Michael	Department of Molecular Neuroscience, Institute of Neurology, University College London, London, UK	Site investigator
Manso, Katarina	Ataxia Centre, Department of Movement and Clinical Neurosciences, Institute of Neurology, University College London, London, UK	Research nurse
Thomas-Black, Gilbert	Ataxia Centre, Department of Movement and Clinical Neurosciences, Institute of Neurology, University College London, London, UK	Site investigator
Garcia-Moreno, Hector	Ataxia Centre, Department of Movement and Clinical Neurosciences, Institute of Neurology, University College London, London, UK	Site investigator
Solanky, Nita	Ataxia Centre, Department of Movement and Clinical Neurosciences, Institute of Neurology, University College London, London, UK	Site investigator
Abeti, Rosella	Ataxia Centre, Department of Movement and Clinical Neurosciences, Institute of Neurology, University College London, London, UK	Site investigator
Polke, James	Laboratory of Neurogenetic The National Hospital for Neurology and Neurosurgery NHNN/UCLH Queen Square WC1N 3BG London UK	Site investigator
Labrum, Robin	Laroratory of Neurogenetic The National Hospital for Neurology and Neurosurgery NHNN/UCLH Queen Square WC1N 3BG London UK	Site investigator
Rodríguez de Rivera Garrido, Francisco Javier	Reference Unit of Hereditary Ataxias and Paraplegias, Department of Neurology, IdiPAZ, Hospital Universitario La Paz, Madrid, Spain	Principle investigator
Mascias, Javier	Reference Unit of Hereditary Ataxias and Paraplegias, Department of Neurology, IdiPAZ, Hospital Universitario La Paz, Madrid, Spain	Site investigator
Sánchez Velasco, Sara	Reference Unit of Hereditary Ataxias and Paraplegias, Department of Neurology, IdiPAZ, Hospital Universitario La Paz, Madrid, Spain	Site investigator
Secades García, Sergio	Reference Unit of Hereditary Ataxias and Paraplegias, Department of Neurology, IdiPAZ, Hospital Universitario La Paz, Madrid, Spain	Site investigator
Mariotti, Caterina	Unit of Medical Genetics and Neurogenetics, Fondazione IRCCS Istituto Neurologico Carlo Besta, Milan, Italy	Principal investigator
Nanetti, Lorenzo	Unit of Medical Genetics and Neurogenetics, Fondazione IRCCS Istituto Neurologico Carlo Besta, Milan, Italy	Site investigator
Castaldo, Anna	Unit of Medical Genetics and Neurogenetics, Fondazione IRCCS Istituto Neurologico Carlo Besta, Milan, Italy	Site investigator
Mongelli, Alessia	Unit of Medical Genetics and Neurogenetics, Fondazione IRCCS Istituto Neurologico Carlo Besta, Milan, Italy	Site investigator
Fichera, Mario	Unit of Medical Genetics and Neurogenetics, Fondazione IRCCS Istituto Neurologico Carlo Besta, Milan, Italy	Site investigator
Klopstock, Thomas	Department of Neurology, Friedrich Baur Institute, University Hospital of the Ludwig-Maximilians-Universität München; German Center for Neurodegenerative Diseases (DZNE), Munich; Munich Cluster for Systems Neurology (SyNergy), Munich, Germany	Principal investigator
Karin, Ivan	Department of Neurology, Friedrich Baur Institute, University Hospital of the Ludwig-Maximilians-Universität München, Munich, Germany	Site investigator
Stendel, Claudia	Department of Neurology, Friedrich Baur Institute, University Hospital of the Ludwig-Maximilians-Universität München, Munich, Germany	Site investigator
Radelfahr, Florentine	Department of Neurology, Friedrich Baur Institute, University Hospital of the Ludwig-Maximilians-Universität München, Munich, Germany	Site investigator
Durr, Alexandra	Institut du Cerveau et de la Moelle épinière, Sorbonne Université, INSERM U 1127, CNRS UMR 7225 and APHP, Pitié-Salpêtrière University Hospital, Genetic Department, Paris, France	Principal investigator
Biet, Marie	Institut du Cerveau et de la Moelle épinière, Sorbonne Université, INSERM U 1127, CNRS UMR 7225	Study coordinator
Charles, Perrine	Pitié-Salpêtrière University Hospital, Paris, France	Site investigator
Ewenczyk, Claire	Pitié-Salpêtrière University Hospital, Paris, France	Site investigator
Just, Jennifer	Department of Neurodegenerative Diseases, Hertie-Institute for Clinical Brain Research & Center of Neurology, University of Tuebingen, Tuebingen, Germany	Site investigator
Koutsis, Georgios	Department of Neurology Eginition Hospital, National and Kapodistrian University of Athens, Athens, Greece	Principal investigator
Walsh, Richard	NationalAtaxia Clinic, Department of Neurology, Adelaide & Meath Hospital Dublin, incorporating the National Children's Hospital, Dublin, Ireland	Principal investigator
Bertini, Enrico	Department of Neurosciences, Unit of Neuromuscular and Neurodegenerative Disorders, Bambino Gesu' Children's Research Hospital, IRCCS, Rome, Italy	Principal investigator
