# Supplementary material for: NfL and pNfH are increased in Friedreich’s ataxia
Source: J Neurol. 2020 Jan 30;267(5):1420–30. doi: 10.1007/s00415-020-09722-6 (PMC7184046; doi:10.1007/s00415-020-09722-6)
Supplement: Supplementary file 1 — Supplementary file1 (DOCX 132 kb) [file 415_2020_9722_MOESM1_ESM.docx]

Supplementary Figure S1

**Fig. S1 Correlation of serum NfL (a, b) and pNfH (c, d) with age in healthy controls and Friedreich’s ataxia**

NfL, Neurofilament light chain; pNfH, phosphorylated neurofilament heavy chain

Supplementary Figure S2

**Fig. S2 Correlation of serum pNfH with disease severity in Friedreich’s ataxia**

Correlation of serum pNfH levels vs. disease severity in patients with Friedreich’s ataxia that were *a priori* categorized as moderately (SARA 10-20) or severely (SARA 30-40) affected.

pNfH, phosphorylated neurofilament heavy chain; SARA, Scale for the Assessment and Rating of Ataxia

Supplementary Figure S3

**Fig. S3 Correlation of serum pNfH with GAA repeat length of the shorter allele (allele 1, a) and the longer allele (allele 2, b) in Friedreich’s ataxia**

pNfH, phosphorylated neurofilament heavy chain

Supplementary Figure S4

**Fig. S4 Increase of SARA over a period of two years**

**p=0.07. Data represent median and 95% confidence interval

SARA, Scale for the Assessment and Rating of Ataxia
